# The Interplay between Topic Shift and Focus in the Dynamic Construction of Discourse Representations

**DOI:** 10.3389/fpsyg.2017.02184

**Published:** 2017-12-08

**Authors:** Xiaohong Yang, Xiuping Zhang, Cheng Wang, Ruohan Chang, Weijun Li

**Affiliations:** ^1^Key Laboratory of Behavioral Science, Institute of Psychology, Chinese Academy of Sciences, Beijing, China; ^2^Department of Psychology, University of Chinese Academy of Sciences, Beijing, China; ^3^Jiangsu Collaborative Innovation Center for Language Ability, Jiangsu Normal University, Xuzhou, China; ^4^Department of Psychology, Zhejiang University of Technology, Hangzhou, China; ^5^Research Center of Brain and Cognitive Neuroscience, Liaoning Normal University, Dalian, China

**Keywords:** focus, topic shift, facilitation, suppression, discourse comprehension, memory

## Abstract

Previous studies have suggested that focusing an element can enhance the activation of the focused element and bring about a number of processing benefits. However, whether and how this local prominence of information interacts with global discourse organization remains unclear. In the present study, we addressed this issue in two experiments. Readers were presented with four-sentence discourses. The first sentence of each discourse contained a critical word that was either focused or unfocused in relation to a wh-question preceding the discourse. The second sentence either maintained or shifted the topic of the first sentence. Participants were told to read for comprehension and for a probe recognition task in which the memory of the critical words was tested. In Experiment 1, when the probe words were tested immediately after the point of topic shift, we found shorter response times for the focused critical words than the unfocused ones regardless of topic manipulation. However, in Experiment 2, when the probe words were tested two sentences away from the point of topic shift, we found the facilitation effect of focus only in the topic-maintained discourses, but not in the topic-shifted discourses. This suggests that the facilitation effect of focus was not immediately suppressed at the point of topic shifting, but when additional information was added to the new topic. Our findings provide evidence for the dynamic interplay between global topic structure and local salience of information and have important implications on how activation of information fluctuates in mental representation.

## Introduction

The comprehension of a discourse involves constructing a mental representation of a discourse. As a discourse unfolds, the representation of the discourse is updated and keeping track of this information is essential for successful discourse comprehension. Given the limitation of cognitive resources, not all information in a discourse is fully attended to. Typically, salient or important information is more fully attended to than other information. One factor that marks the salience of information is linguistic focus.

Focus has often been defined as the most prominent information in a sentence (Halliday, 1967). There are several ways to mark focus in context. One typical way to mark focus is question–answer pairs. In question–answer pairs, the constituent that provides an answer to a question is focused. For example, in the conversation *Who did Mary vote for?/Mary voted for [JOHN]*, JOHN supplies an answer to the preceding question. Hence, JOHN is focused (Jasinskaja et al., 2004). Besides question-answer pairs, focus can also be realized by placing pitch accent on the focused element, by using cleft-structures (e.g., *it was*… who/that…), or by using focus-particles (e.g., *only, always*).

In the field of psycholinguistics, the effect of focus in online sentence/discourse comprehension has been an important issue that received considerable attention. Numerous studies have shown that focus can produce a number of processing benefits. Firstly, focused items receive more attention and are processed more quickly than unfocused items (Cutler and Fodor, 1979; Morris and Folk, 1998; Chen et al., 2012, 2014; Kristensen et al., 2013; Wang et al., 2013). For instance, reaction time to detect a phoneme target in a sentence was found to be faster when the word in which the target occurred formed part of the semantic focus of the sentence (Cutler and Fodor, 1979). Furthermore, focus leads to more detailed lexical semantic processing and enhanced memory representations of information (Bredart and Modolo, 1988; Birch and Rayner, 1997; Cutler and Clifton, 1999; Klin et al., 2004; Sturt et al., 2004; Ward and Sturt, 2007; Wang et al., 2009, 2011). For instance, when a word changed, participants were more successful at detecting it when it was in focus than when it was not in focus, indicating that focusing an element led to more detailed memory representation (Ward and Sturt, 2007).

These studies on focus thus provide evidence that focusing an element can bring about a facilitation effect for the focused element. A general issue for these studies is that the effect of focus is always examined without the consideration of global discourse variables. In fact, one particular aspect for any natural discourse is that its subunits can form various structures, which can affect the activation of information in a discourse (Hyönä, 1995; Yang et al., 2013, 2014). Given that focus information generally appears in continuous discourse and that discourses vary in internal structures, the question that is left answered is whether variations in global discourse structure can modulate the effect of focus. Knowledge about this question can have important implications on how the activation of information fluctuates in continuous discourses.

According to the structure building framework, a classical theory of discourse comprehension, information that triggers comprehenders to shift can produce a suppression effect on discourse information (Gernsbacher, 1997). This framework proposes that to build a coherent mental representation, at least three stages are involved (Gernsbacher, 1997). At the initial stage of laying a foundation, readers lay foundation for their mental structures. The building blocks for the mental structures are memory nodes which are activated by incoming stimuli. For instance, when readers encounter the first sentence of a discourse “*Xiao Wang is a very kind man*,” they may activate the memory nodes for *Xiao Wang, kind*, and *man.* This stage of laying mental foundations has been evidenced by the fact that comprehenders slowed down when reading the first sentence of a paragraph (Haberlandt and Graesser, 1985) and concepts mentioned first in an episode were accessed faster than concepts mentioned later (Kim et al., 2004). Once a foundation is laid, the second stage, i.e., the mapping stage begins when readers gradually develop the representation by mapping on new information. Thus, when readers continue to read the second sentence of the discourse “*This year he has diabetes*,” they may attach the information *This year* and *diabetes* to *Xiao Wang.* This mapping process was evidenced by the observation that readers actively use pronouns, definite articles, and conceptual anaphora in establishing coherence (Foertsch and Gernsbacher, 1997; Ferstl and von Cramon, 2001). The mapping stage can go on smoothly until incoming information indicates a change. In this case, the third stage, i.e., the shifting stage begins and readers shift from building one substructure to develop another. When comprehenders shift to build a new substructure, the old substructure can be suppressed and the mapping stage restarts when the information related to the new substructure is continuously mapped onto it. Thus, if incoming information shifts from *Xiao Wang* to *His wife* such as in “*His wife has diabetes*,” then readers may suppress the structure about *Xiao Wang* and build a new substructure for *His wife.* This could become more evidenced when more information about *His wife* but not *Xiao Wang* is read.

Then, what can trigger comprehenders to shift during discourse processing? In a continuous discourse, a sentence may shift or continue the topic of the previous sentence (Givon, 1983; Lorch et al., 1985, 1987; Hyönä, 1994, 1995; Oberlander, 2004; Smith, 2004). Previous studies have found that changes in topic can trigger comprehenders to shift and build a new substructure (O’Brien et al., 1986; Binder and Morris, 1995). This was evidenced by the fact that a sentence that shifts the topic of the preceding sentences can bring about increased processing time than one that continues the topic of the preceding sentences (Lorch et al., 1985, 1987; Hyönä, 1994; Hyönä and Lorch, 2004). Furthermore, recent ERP studies have shown that topic shifts consistently elicited a late positivity effect, which was interpreted as reflecting the process of discourse updating caused by shifting (Hirotani and Schumacher, 2011; Hung and Schumacher, 2012; Yang et al., 2013).

Apart from triggering readers to shift and build a new substructure, topic shifts can also produce a suppression effect on the old mental structure. Studies on anaphoric inferences and ambiguity resolution have suggested a suppression effect of topic shifts such that when encountering topic shifts, information related to the earlier topic is faded or is less available (O’Brien et al., 1986; Binder and Morris, 1995). For instance, in Binder and Morris (1995), researchers monitored eye movements as participants read passages that contained two occurrence of a balanced ambiguous word. For instance, the first occurrence of the target word *fans* was in “*Several hours before the performance the arena was filled with screaming fans*” and the second encounter of the target word *fans* was in “*There was no air conditioners or fans set up to keep people comfortable*” in the topic maintained condition or “*the blowing fans were not working properly*” in the topic shifted condition. It was found that repeating an ambiguous word and changing its meaning across the two occurrences produced a reading disadvantage, but only when the topic between the first and second encounter of the ambiguous word remained the same (e.g., *the concert*) throughout the passage. When there was a topic shift (shifted from *the concert* to *the kitchen*), this disadvantage disappeared. This was interpreted as suggesting that topic shifts suppressed the old discourse representation so that integration difficulty resulting from a change in the meaning of the ambiguous words disappeared.

Thus, in light of the structure building framework and related evidence, topic shifts can be of particular relevance during discourse comprehension and most probably they would reduce the accessibility of information presented before the topic shifts. Given that focus produces a facilitation effect and topic shift produces a suppression effect, it is reasonable to speculate that these two internal components of discourse might interact with each other and affect discourse comprehension. Therefore, our first question was to examine whether there is interplay between these two factors during discourse comprehension.

Focus structure and topic structure for Chinese has its peculiarity as well as general characteristics with other Western Germanic languages. For focus structure, Chinese uses more syntax and less phonology in focus realization compared with Western Germanic languages (i.e., English) (Xu, 2004). The pragmatic notion of focus (mainly informational focus) is syntactically encoded to a larger extent in Chinese than in many other languages. Meanwhile, it has been shown that in Chinese, speakers and writers have the tendency to place new constituents, which receive informational focus, late in the sentence (Chao, 1968; LaPolla, 1995; Xu, 2004). As for topic structure, Chinese is a topic-prominent language in the terms of Li and Thompson (1976). It means that topic always appears at the initial position of the sentence and represents what the following information of the sentence is about. Therefore, Chinese readers/listeners have the potential tendency to predict focus and topic to be present at different positions of a sentence. These characteristics make it easier to segregate the effects of focus and topic in experimental design and observe their interaction. Moreover, as focus is a local phenomenon while a topic frequently organize several sentences or even a whole discourse in Chinese (Li, 2004), examining their interaction can reveal how readers make use of local prominence and global discourse organization during discourse comprehension.

If focus and topic shift interact to influence mental representations, a related question then arises as to at which point can the interaction be expected. As specified by the structure building framework, when encountering topic shifts, readers develop a new substructure and suppress the old one. Then readers move on pass the shifting stage and start to map incoming information onto the new substructure. Although topic shifts can produce a suppression effect on the content of the old topic (O’Brien et al., 1986; Binder and Morris, 1995), we do not know whether it could immediately suppress the focused information of the old topic given that focus can produce a very robust effect on the focused element (e.g., Bredart and Modolo, 1988; Birch and Rayner, 1997; Cutler and Clifton, 1999; Klin et al., 2004; Sturt et al., 2004; Wang et al., 2011). If the effect of focus is immediately suppressed at the point of topic shifting, we might expect to find the interaction between topic and focus at the shifting stage. However, if the effect of focus is not suppressed until more incoming information is added to the new substructure such that it becomes demanding for the working memory system to maintain a high level of activation for the focused information of the old topic, then we might expect to find the interaction when readers move on pass the shifting point and start to add incoming information onto the new substructure.

To address the issues mentioned above, we conducted two experiments in which a probe-recognition task was used to test the memory representation of the critical words. Four conditions were created depending on whether the critical word in the first sentence of each discourse was focused or unfocused and whether the second sentence of the discourse shifted or maintained the topic of the first sentence, namely: topic-shifted/focused, topic-shifted/unfocused, topic-maintained/focused, and topic-maintained/unfocused (see **Table 1** for an example stimulus).

**Table 1:** Example stimuli used in Experiment 1.

Lead-in question: focused question/unfocused question
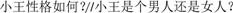
What is the personality of Xiao Wang?/Is Xiao Wang a man or a woman?
First sentence
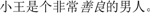
Xiao Wang is a very *kind* man.
Second sentence: topic-shifted condition/topic-maintained condition
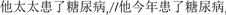
His wife has diabetes,/this year he has diabetes,
Third sentence:
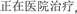
and is currently being treated in hospital,
Final sentence:
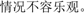
The situation is not optimistic.
Probe:
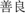
Kind


*Translations are presented below each Chinese sentence. Target words are marked in italics.*

In Experiment 1, the probe words were placed at the end of the second sentence (that is, the topic shift point) to test whether an interaction between topic structure and information structure could be found when readers encountered a topic shift. In Experiment 2, the probe words were placed at the end of the fourth sentence of each discourse which was far away from the point of topic transition to test whether an interaction effect could be found when readers moved on past the shifting point.

## Experiment 1

In Experiment 1, we aimed to test whether there would be a modulation effect of discourse topic on local information salience when readers encountered a topic shift. Thus, we test the accessibility of the critical words immediately after the presentation of the topic-shifted sentences. Based on previous findings suggesting that focusing an element leads to a facilitation effect on memory representation of the element (Cutler and Fodor, 1979; Ward and Sturt, 2007), we expected to find the facilitation effect of quicker responses and higher accuracy rates for the focused items compared with the unfocused ones. Furthermore, given that topic shifts could produce a suppression effect (Anderson et al., 1983; O’Brien et al., 1986; McKoon et al., 1993; Binder and Morris, 1995; Yang et al., 2013), we expected that the facilitation effect of focus could only be found in the topic-maintained condition, but not in the topic-shifted condition.

### Method

#### Participants

Forty-eight college students (18 males; mean age = 22.9 years; *SD* = 2.2 years) volunteered to take part in the Experiment. All were native speakers of Chinese and were paid for their participation.

#### Materials

Originally Eighty four-sentence discourses were constructed. Each set of discourses was written in both topic-shifted and topic-maintained conditions. Each discourse began with a sentence that introduced a topic. This sentence was followed by the second sentence which either changed (topic-shifted condition) or maintained (topic-maintained condition) the topic of the first sentence. Then, two more sentences ended the discourse. These two sentences were identical across both conditions. Our definitions of topic were based on the commonly held beliefs about the features of topic in Chinese: (1) a topic is an entity that is introduced at the initial position of a sentence, (2) a topic is always followed by a comment (Li and Thompson, 1976; Li, 2004). A topic shift was thus operationalized as introducing a new entity at the initial position of the second sentence of a discourse. Note that while focus projects constituent prominence at sentence level, topic in the present study is less of a sentential phenomenon, but more of a discourse phenomenon (i.e., discourse topic) because the topics in our materials were used to organize several sentences and represent what these sentences were about rather than only represent the proposition as to what information is given at a sentence (Binder and Morris, 1995). Specifically, in the topic-maintained condition, each discourse as a whole was organized under a single topic while in the topic-shifted condition, the new topic introduced in the second sentence was used to organize the following stretch of the discourse.

To control for discourse coherence as a confounding factor of topic manipulation, we conducted a rating study in which 24 participants who did not participate in the formal experiments reported here were asked to rate the coherence of the discourses on a five-point scale ranging from 1 (not coherent) to 5 (very coherent). Different conditions of the discourses were distributed to different lists so that each participant saw only one condition of a given discourse. On the basis of the rating scores, 40 sets of discourses were selected for the main study so that the coherence rating scores were matched between the topic-shifted and the topic- maintained condition [*t*(23) = -0.643, *p* > 0.05, mean ± SD = 3.35 ± 0.79; 3.42 ± 0.73; respectively for the topic-shifted and the topic- maintained condition].

As shown in **Table 1**, to manipulate focus, we added a wh-question before each discourse and focus was operationalized as the constituent that supplied an answer to the preceding wh-question (Jasinskaja et al., 2004; Wang et al., 2009, 2011). Two versions of the wh-question were constructed (focused context and unfocused context). For the focused condition, we used a focused question context so that the critical word in the first sentence provided an answer to the preceding question. In contrast, for the unfocused condition, we used an unfocused question context so that the critical word in the first sentence did not provide an answer to the preceding question. These critical words were later used as probe words in the probe recognition task. By combining focus manipulation and topic manipulation, four conditions were created.

Using a Latin square design, the 40 selected items were then separated into four lists so that one version of each item appeared on each list. In the experimental materials, the probe words were always the critical words that had appeared in the discourses and required a “Yes” answer for the probe recognition task. Therefore, to each list we also added 40 fillers in which the probe words required a “No” answer so as to balance the “Yes” or “No” responses. An example filler discourse is given in **Table 2**.

**Table 2:** Example filler discourse used in the present study.

Lead-in question: focused question/unfocused question
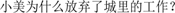
Why did Xiao Mei give up her job in the city?
First sentence
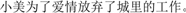
Xiao Mei gave up her job in the city for love.
Second sentence:
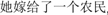
She married a farmer,
Third sentence:
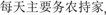
and every day she takes care of the farm and her family,
Final sentence:
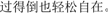
She feels easy and comfortable.
Probe:
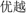
superior


*Translations are presented below each Chinese sentence.*

#### Procedure

Each participant was assigned one of the four lists of materials. Each session began with six practice discourses to ensure that the participants were familiarized with and understood the procedure. Then the stimuli were presented in two blocks, which were separated by a short break. Each trial began with a fixation (lasted 1000 ms) in the middle of the screen. Then a wh-question appeared on the screen for another 2000 ms. After that the four sentences of the discourses were presented sentence by sentence. The participants were instructed to read each discourse sentence by sentence at their normal speed. They were told to press the space bar to end the current sentence and present the next sentence. The probe words of interest appeared immediately after the topic-shifted sentences (i.e., the second sentence of the discourses). Participants were told to judge whether or not the word had appeared in the first two sentences by pressing the J (for “Yes” response) or F (for “No” response) key in the keyboard. The maximum display time for the probe words was 5 s. Once the participants made their response, the display of the probe words terminated and the third sentence of the discourse appeared on the screen. They also received a comprehension question (for 1/2 of the trials) at the end of the discourse and they had to make a quick judgment. They again used the J or F key in the keyboard. These comprehension questions were used to ensure that readers would read attentively for comprehension.

### Results and Discussion

For the comprehension task, one participant had an accuracy rate of lower than 70%. Thus, this participant was excluded from further statistical analysis. The remaining participants achieved an accuracy rate of 91% on average (mean ± SD = 0.88 ± 0.15, 0.89 ± 0.14, 0.91 ± 0.12, and 0.94 ± 0.11 for the topic-shifted/focused, topic-shifted/unfocused, topic-maintained/focused, and topic-maintained/unfocused condition respectively), indicating that they indeed attended to the materials.

For the analysis of the probe-recognition task, trials with response times longer than 3000 ms or without a response were eliminated (Goschke and Kuhl, 1993). Reaction times that were less than or more than 2.5 SD of the subject mean were also removed from analysis (Selst and Jolicoeur, 1994). These procedures removed 5.4% of all data. Mean accuracy rates and reaction times are shown in **Table 3**. The data were analyzed using a linear mixed effects model (LMM) (Bates, 2005; Baayen et al., 2008) that included fixed effects of focus (focused vs. unfocused), topic shift (topic-shifted vs. topic-maintained), and by-participant and by-item random intercepts with the free software R. Rating scores of discourse coherence were also added as a covariate in the analysis to control for its effects on the dependent variables. The lmer() function of the lmer4 package was used to estimate fixed effects and parameter estimation of the LMM. The degree of freedom and *p*-values were computed using anova() function of the lmerTest package with Satterthwaite approximations. The LMM estimates of the fixed effects were provided in the **Supplementary Materials**.

**Table 3:** Results for the probe-recognition task in Experiment 1 (with standard errors in parentheses).

Measures (ms)	Topic-shifted		Topic-maintained	
		
	Focused	Unfocused	Focused	Unfocused
Reaction times	1059 (33)	1096 (30)	1019 (28)	1093 (32)
Accuracy rates	0.95 (0.01)	0.93 (0.01)	0.96 (0.01)	0.92 (0.02)


For reaction times, we found a significant main effect of focus [*F*(1,1634.95) = 21.38, *p* < 0.001]. The trend was for the focused condition to yield shorter reaction times than the unfocused condition. Neither the main effect of topic shift nor the interaction between topic shift and focus was significant (*F*s < 2.56, *ps* > 0.05). A parallel analysis was conducted on the errors. A binomial family was used because of the binary nature of the responses. None of the fixed effects were significant (|*z*|*s* < 1.43, *ps* > 0.05).

Our results showed that focusing an element brought about faster response times, which were consistent with previous studies that found a facilitation effect of focus (Ward and Sturt, 2007; Wang et al., 2009, 2011). However, the crucial hypothesis tested in this experiment was not confirmed, since we did not find an interaction between topic shift and focus when readers encountered a topic shift. At the point of topic shifting, although readers shifted to build a new substructure for the new topic, there was only limited information attached to the new topic. Therefore, it could be possible that at this point, the facilitation effect of focus for the old topic was still robust and the focused items were still highly activated in working memory. It is therefore interesting whether the facilitation effect of focus of the old topic could persist into memory as more information was attached to the new topic. This issue was addressed in Experiment 2.

## Experiment 2

Experiment 2 aimed to explore whether there was an interaction between topic shift and focus when readers moved on past the shifting point and more information was attached to the new topic. To investigate this issue, we moved the probe words to the discourse final position which was two-sentence away from the topic shift point. We expected that as more information was attached to the new topic, it would become more demanding for the working memory system to hold the focused information of the previous topic at a high activation level. Thus, an interaction effect between focus and topic shift should be observed such that the facilitation effect of focus was eliminated in the topic-shifted discourses.

### Method

#### Participants

Forty-eight college students (20 males; mean age = 23.8 years; *SD* = 2.5 years) were recruited to participate in the experiment. They were paid for their participation. All were native speakers of Chinese, and none had participated in the previous experiment or pretest.

#### Materials and Procedure

The materials were the same as in Experiment 1. The procedure was the same with Experiment 1, with one exception: The probe words were moved to the end of the discourses. As in the previous experiment, we added some comprehension questions to ensure that readers would read attentively for comprehension. The probe questions always preceded the comprehension questions.

### Results and Discussion

For the comprehension task, two participants had an accuracy rate of lower than 70%. Thus, these two participants were excluded from further statistical analysis. The remaining participants achieved an accuracy rate of 88% on average (mean ± SD = 0.80 ± 0.17, 0.86 ± 0.16, 0.94 ± 0.11, and 0.93 ± 0.11 for the topic-shifted/focused, topic-shifted/unfocused, topic-maintained/focused, and topic-maintained/unfocused condition respectively), indicating that they did attend to the materials.

For the probe recognition task in Experiment 2, the criterions for outlier trimming used in Experiment 1 were again used for RT analysis. 6.5% of all data were removed. The reading times and accuracy rates are presented in **Table 4**. As with Experiment 1, data were processed using a LMM.

**Table 4:** Results for the probe-recognition task in Experiment 2 (with standard errors in parentheses).

Measures (ms)	Topic-shifted		Topic-maintained	
		
	Focused	Unfocused	Focused	Unfocused
Reaction times	1212 (47)	1238 (42)	1174 (38)	1283 (49)
Accuracy rates	0.93 (0.02)	0.89 (0.02)	0.93 (0.01)	0.87 (0.02)


For reaction times, there was a significant main effect of focus [*F*(1,1516.4) = 17.08, *p* < 0.001]. The trend was for the focused condition to yield shorter reaction times than the unfocused condition. More importantly, there was a significant focus by topic shift interaction [*F*(1,1517.7) = 5.10, *p* < 0.05]. Planned comparisons showed that participants were faster to recognize a probe word when the word was in focus position than when it is outside the scope of focus in the topic-maintained discourses [*t*_(729.1)_ = 4.38, *p* < 0.001], but not in the topic-shifted discourses [*t*_(722.3)_ = 1.48, *p* > 0.1].

A parallel analysis was conducted on the errors. A binomial family was used because of the binary nature of the responses. There was again a significant main effect of focus (*Z* = -2.42, *p* < 0.05). Participants were more able to make accurate responses when the probe word was focused than when it was unfocused. Neither the main effect nor the interaction between focus and topic shift were significant (|*z*|s < 0.39, *ps* > 0.1).

These results again showed a processing advantage for the focused over unfocused words: readers were faster and more accurate at identifying the focused probe words. More importantly, an interaction effect between focus and topic shift was found, which clearly showed that the facilitation effect of focus was eliminated by the suppression effect of topic shift when more information was attached to the new topic. Specifically, focusing an item facilitated information retrieval only in the topic-maintained discourses, but not in the topic-shifted discourses. This provides clear evidence that topic shifts modulate the facilitation effect of focus during online construction of discourse representation.

## General Discussion

The goals of the present study were to determine whether and how the facilitation effect of focus was modulated by discourse structure. The results of Experiment 1 showed that the facilitation effect of focus was not immediately suppressed when upcoming sentences indicated a change in topic. However, in Experiment 2, we found that after reading additional sentences, the interaction between topic shift and focus appeared. These results suggest that the mental representation of a discourse involves dynamic interplay between the organization of discourse topic and the prominence of local information.

### The Facilitation Effect of Focus

Across experiments, we found that focusing an element produced a facilitation effect. Focused probe words were identified more quickly and more accurately than unfocused words. This agrees with previous studies that words were consistently better remembered when they had been focused (Sturt et al., 2004; Ward and Sturt, 2007). This facilitation effect of focus can be a result of attention allocation. It has been shown that readers have longer reading times when reading a region of the sentence that was focused than when reading a region that was not focused, which suggests that readers encode focused information more carefully (Birch and Rayner, 1997). Moreover, recent electrophysiological research has found that focus processing is associated with the P2 component, which is generally regarded as an index of attention allocation (Chen et al., 2014). In the current study, the questions preceding the discourses explicitly marked focus position, which could have served as mental instructions and informed readers about the salience of information. Thus, upon reading the focus words, more attention resources were allocated to them. Alternatively, the processing advantage of focus could also be a facilitation of retrieving information from working memory, that is, a benefit at retrieval/probe. It has been suggested that while retrieval operations are required for unfocused information, information in focal attention is actively maintained in working memory and does not need to be retrieved before being bought to bear on on-going operations (Foraker and McElree, 2011). Thus, a retrieval benefit could be contributing to our results.

The finding of the facilitation effect of focus in Experiment 2 is noteworthy. In this experiment, the focus effect was tested with three intervening sentences that separated the manipulation of focus and the probe word. This suggests that the facilitation effect of focus is not transient, but can persist into memory as discourse unfolds. In previous studies, the effect of focus was mostly tested at the point of focus manipulation or immediately after it (Ward and Sturt, 2007; Chen et al., 2012, 2014; Chen and Yang, 2015). Thus, they provided little information about whether the effect of focus was quickly lost from readers’ memory. Our data, however, have provided strong evidence for the persistence of the focus effect.

### Influence of Topic Shift on the Facilitation Effect of Focus

The crucial hypothesis tested in the present study was whether and how focus interplayed with topic shift to influence mental representation. In Experiment 1, no interaction was observed when the probe words were placed immediately after the topic shift sentences. In Experiment 2, however, we found an interaction between focus and topic structure: The facilitation effect of focus was only found in topic-maintained condition, but not in topic-shifted condition. These results suggest that discourse structure can override local information salience and agree with previous findings that the processing of topic shifts can produce a suppression effect (Binder and Morris, 1995; Yang et al., 2013).

Note that in Experiment 2 in which the interaction was found, the probe words were placed after two more sentences which elaborated on the new topic. According to the structure building framework, when encountering a topic shift, comprehenders shift to build a new substructure for the new topic and once the foundation for the new topic is laid, the mapping stage begins when readers gradually develop the representation by mapping on new information (Gernsbacher, 1997). Thus, our results could be an indication that the interaction did not take place at the shifting stage, but at the following mapping stage.

The observed suppression effect of topic shifts on focus could be due to the allocation of cognitive resources. According to the structure building framework, the introduction of a new topic can trigger readers to shift and build a new substructure while at the same time suppress the structure of the old topic (Gernsbacher, 1997). However, given that generally readers allocate more cognitive resources to the processing of focused elements and that at the shifting stage, the new substructure of the new topic only included very limited information, therefore, it could be possible that at this stage, readers were still able to hold the focused information of the old topic and the new substructure of the new topic in working memory. Thus, at this stage, no interaction was observed. However, when readers moved on past the shifting point and continued to read additional information about the new topic, the mental representation of the new topic continued to be developed. This could capture more cognitive recourses and reduce the recourses allocated to the processing of focus. There could be two consequences for this reduction of cognitive resources. Readers might actively suppress the activation of the old topic or there could be a more passive loss of activation or lesser degree of availability in working memory for the old topic. In either case, the structuring of focus of the old topic could disappear. Therefore, the suppression effect of topic shifts on focus was observed.

The presence and absence of the interaction effect observed in the current study have important theoretical implications: The mental construction of discourse representation involves dynamic interplay between discourse organization and local information structure. How the mental representation of a discourse is constructed has been a central issue for most theories of discourse comprehension. There are theoretical descriptions on the role of discourse organization such as topic shifts (Gernsbacher, 1997) and event boundaries (Zwaan et al., 1995a; Van den Broek et al., 2005). Furthermore, several theories have emphasized how global discourse context influences local coherence processing (Fletcher and Bloom, 1988; Glenberg and Langston, 1992; McKoon and Ratcliff, 1992; Albrecht and O’Brien, 1993). Local coherence involves connecting upcoming words with immediate preceding information in a sentence while global coherence involves integrating upcoming words with a much wider discourse context. Some theories have claimed that readers used global context only when there is a local coherence break (Fletcher and Bloom, 1988; McKoon and Ratcliff, 1992) while others argued that readers attempted to establish coherence at both local and global level (Glenberg and Langston, 1992; Albrecht and O’Brien, 1993). These theoretical discussions have advanced our understanding on discourse comprehension. However, how local prominence of information, rather than local coherence, interacts with discourse organization has rarely been discussed. This issue is directly related to our understanding of how activation of information fluctuates in mental representation. Given the interaction observed in the current study, we propose that this issue should be incorporated into future models of discourse comprehension.

Shifts in topic is only one kind of shifts and there are also other shifts in a discourse that can trigger readers to shift from current representation and build a new substructure (Zwaan et al., 1995b; Gernsbacher, 1997; Speer and Zacks, 2005), such as time (e.g., *a moment later* vs. *1 year later* as in Ditman et al., 2008) and location (e.g., remaining *in the classroom* vs. *went to the bar* as in Levine and Klin, 2001). Previous studies have suggested that different kinds of narrative shifts function differently (Rich and Taylor, 2000). One important direction for future research, therefore, will be to explore whether shifts in other discourse dimensions, such as time and location, will have a different modulation effect on local information structure. A related issue is whether shifts in two or more dimensions will have a more immediate effect on local salience of information. There is some evidence supporting that during the comprehension of continuous text, shifts are largely processed separately in a way consistent with incremental updating and may have combined effects (Curiel and Radvansky, 2014). Thus, it could be possible that combined effect of different kinds of shifts will require more attention resources and exert more immediate effect on local information structure.

## Conclusion

To conclude, in two experiments, we found that the facilitation effect of focus could still function when readers encountered topic shifts, but could be suppressed when additional information was added to the new topic. These results suggest that discourse organization can override local information structure. Our findings provide evidence for a dynamic relationship between discourse topic organization and local salience of information and highlight the need to consider their interaction for the model of discourse comprehension.

## Ethics Statement

This study was approved and carried out in accordance with the recommendations of the Institutional Review Board of the Institute of Psychology, Chinese Academy of Sciences with written informed consent from all subjects. All participants provided written, informed consent before taking part in our experiments.

## Author Contributions

XY: experiment design, manuscript writing. XZ and RC: data collection, data analysis. CW: data analysis, manuscript writing. WL: manuscript writing.

## Conflict of Interest Statement

The authors declare that the research was conducted in the absence of any commercial or financial relationships that could be construed as a potential conflict of interest.

## Supplementary Material

**DATA SHEET 1 |** Experimental stimuli.
