# Supplementary material for: The Interplay between Topic Shift and Focus in the Dynamic Construction of Discourse Representations
Source: Front Psychol. 2017 Dec 8;8:2184. doi: 10.3389/fpsyg.2017.02184 (PMC5727373; doi:10.3389/fpsyg.2017.02184)
Supplement: Supplementary file 2 [file Presentation_1.PDF]

LMM analyses

Experiment 1 RTs

```
>
> RT.lmer5=lmer(RT~coherence+topic+focus+topic:focus +(1|subject)+(1|item), data.RT)
>
> anova(RT.lmer5)
Analysis of Variance Table of type III  with  Satterthwaite
approximation for degrees of freedom
             Sum Sq Mean Sq NumDF   DenDF F.value    Pr(>F)
coherence     23767   23767     1   81.95  0.3541    0.5534
topic        171928  171928     1 1663.60  2.5617    0.1097
focus       1435243 1435243     1 1634.95 21.3846 4.053e-06 ***
topic:focus   61405   61405     1 1635.08  0.9149    0.3390
---
Signif. codes:  0 ‘***’ 0.001 ‘**’ 0.01 ‘*’ 0.05 ‘.’ 0.1 ‘ ’ 1
> |
```

Table 1 | LMM estimates of fixed effects for RTs in Experiment 1.

Fixed effects	Measure		
	Estimate	Std. Error	t value
(Intercept)	1079.36	69.19	15.60***
Coherence	-10.88	18.28	-0.60
Topic	-32.23	17.60	-1.83
Focus	45.97	17.71	2.60**
Topic : focus	24	25.1	0.96

Note: *** p <.001, ** p <.01, * p <.05.

Experiment 1 ACC

Table 2 | LMM estimates of fixed effects for ACC in Experiment 1.

Fixed effects	Measure		
	Estimate	Std. Error	Z value
(Intercept)	4.04	0.68	5.96***
Coherence	-0.22	0.18	-1.20
Topic	0.25	0.33	0.75
Focus	-0.41	0.29	-1.43
Topic : focus	-0.38	0.42	-0.90

Note: *** p <.001, ** p <.01, * p <.05.

Experiment 2 RTs

```
> RT.lmer5=lmer(RT~coherence+topic+focus+topic:focus +(1|subject)+(1|item), data.RT)
>
> anova(RT.lmer5)
Analysis of Variance Table of type III  with  Satterthwaite
approximation for degrees of freedom
             Sum Sq Mean Sq NumDF  DenDF F.value    Pr(>F)
coherence     14506   14506     1   62.8  0.1430   0.70662
topic          2429    2429     1 1535.7  0.0239   0.87706
focus       1732784 1732784     1 1516.4 17.0780 3.784e-05 ***
topic:focus  517292  517292     1 1517.7  5.0983   0.02409 *
---
```

Table 3 | LMM estimates of fixed effects for RTs in Experiment 2.

Fixed effects	Measure		
	Estimate	Std. Error	t value
(Intercept)	1166.91	76.71	15.21***
Coherence	7.11	18.80	0.38
Topic	-38.59	22.29	-1.73
Focus	29.92	22.63	1.32
Topic : focus	72.21	31.98	2.26*

Note: *** $p < .001$, ** $p < .01$, * $p < .05$.

Experiment 2 RTs

Multiple comparison

Table 4 | LMM estimates of fixed effects for RTs in the topic-shifted discourses.

Fixed effects	Measure		
	Estimate	Std. Error	t value
(Intercept)	1178.12	104.94	11.23***
Coherence	3.54	28.24	0.13
Focus	31.39	21.99	1.43

Table 5 | LMM estimates of fixed effects for RTs in the topic-maintained discourses.

Fixed effects	Measure		
	Estimate	Std. Error	t value
(Intercept)	1157.67	74.92	15.45***
Coherence	-1.88	18.25	-0.10
Focus	101.43	23.16	4.38***

Experiment 3 ACC

Table 6 | LMM estimates of fixed effects for ACC in Experiment 2.

Fixed effects	Measure		
	Estimate	Std. Error	Z value
(Intercept)	3.29	0.60	5.44***
Coherence	-0.12	0.17	-0.72
Topic	0.06	0.26	0.23
Focus	-0.57	0.23	-2.42*
Topic : focus	-0.13	0.33	-0.39

Note: *** $p < .001$, ** $p < .01$, * $p < .05$.
